# Is abdominal drainage after open emergency appendectomy for complicated appendicitis beneficial or waste of money? A single centre retrospective cohort study

**DOI:** 10.1016/j.amsu.2018.10.040

**Published:** 2018-11-09

**Authors:** Ahmed Kamel Abdulhamid, Shah-Jalal Sarker

**Affiliations:** a Department of Surgery, Imam Hussein Medical City Hospital, Kerbala Medical University, Kerbala, Iraq; b UCL Medical School, UCL, Research Department of Medical Education, Room GF/664, Royal Free Campus, Hampstead, London, NW3 2PR, UK

**Keywords:** Appendicitis, Complicated, Open appendectomy, Intra-peritoneal abscess, Wound infection, Perforated, Post-operative complications, Abdominal drain

## Abstract

**Background:**

Appendicitis is a medical condition that causes painful inflammation of the appendix. For acute appendicitis, appendectomy is immediately required as any delay may lead to serious complications such as gangrenous or perforated appendicitis with or without localized abscess formation. Patients who had appendectomy for complicated appendicitis are more prone to develop post-operative complications such as peritoneal abscess or wound infection. Sometimes, abdominal drainage is used to reduce these complications. However, the advantage of the abdominal drainage to minimize post-operative complications is not clear. Therefore, the aim of this study was to investigate whether the use of abdominal drainage after open emergency appendectomy for complicated appendicitis (perforated appendicitis with localized abscess formation only) can prevent or significantly reduce post-operative complications such as intra-peritoneal abscess formation or wound infection.

**Methods:**

In this retrospective cohort study, files and notes were reviewed retrospectively for patients who had open emergency appendectomy for complicated appendicitis (perforated appendicitis with localized abscess formation only) and who had already been admitted and discharged from the surgical wards of Kerbala medical university/Imam Hussein medical city hospital/Kerbala/Iraq. Patients were selected according to specific inclusion and exclusion criteria. Patients were divided into two groups; drainage and non-drainage groups. The drainage group had intra-abdominal drain inserted after the surgery, while the non-drainage group had no drain placed post-operatively. A comparison between both groups was done in terms of these parameters; (i) the development of post operative intra-peritoneal abscess and or wound infection. (ii) The length and cost of hospital stay. (iii) The mortality outcomes. Statistical analysis was done using Pearson Chi-square test, Independent sample *t*-test and Mann-Whitney *U* Test.

**Results:**

Of 227 patients with open emergency appendectomy for complicated appendicitis, 114 had received abdominal drain after the surgery. Fifty out of 114 patients (43.9%) with abdominal drainage developed post-operative intra-peritoneal abscess (abdominal or pelvic) while 53 out of 113 patients (46.9%) without drainage developed the same complication (*P* = 0.65). It was also revealed that for patients with drainage, 42 patients (36.8%) had post-operative wound infection, whereas this number was 38 (33.6%) for patients without drainage (*P* = 0.61). On the other hand, the patients with drain had significantly longer length of hospital stay (mean length of stay: 4.99 days versus 2.12 days, *P* < 0.001) and significantly higher cost (median cost per patient: $120 versus $60, *P* < 0.001).

**Conclusion:**

Installation of abdominal drainage after open emergency appendectomy for complicated appendicitis did not bring any considerable advantage in terms of prevention or significant reduction of post-operative intra-peritoneal abscess and wound infection. Rather, it lengthened the hospital stay and doubled the cost of operation.

## Highlights

• The use of abdominal drain after open appendectomy for complicated appendicitis does not bring any considerable advantage in terms of prevention or significant reduction of post-operative intra-peritoneal abscess and wound infection.
• Insertion of drain lengthens hospital stay and doubles the cost of operation.
• No mortality complications had been identified in both drain and non drain groups.

## 1 Introduction

Right lower abdominal pain is a very common surgical presentation. There are many reasons behind this condition but the most common cause is appendicitis. Appendicitis is infection and inflammation of the appendix [1]. Acute appendicitis requires immediate diagnosis and treatment as any delay may lead to gangrenous or perforated appendicitis with or without localized collection (complicated appendicitis). The patients who had appendectomy for complicated appendicitis are more likely to develop post-operative complications such as intra-peritoneal abscess (abdominal or pelvic) or wound infection [2]. Typically, to prevent these complications abdominal drainage is used.

The incidence of acute appendicitis is about 76–227 patients per 100,000 people per year in different parts of the world [3]. The risk of suffering from it according to study done in United States of America is about 7%–9% [4]. Many studies show that it affects mostly individuals who belong to 10–19 age-group [4].

Acute appendicitis can be classified into two types: complicated and uncomplicated. Complicated acute appendicitis means late or advance stage of infection which might occur due to delay in the presentation or diagnosis or treatment. These complications may be gangrenous appendicitis or perforated appendicitis with or without abscess formation with or without local or general peritonitis [[5], [6], [7]].

Diagnosis of acute appendicitis is usually clinical but sometimes abdominal ultrasound, blood tests and urine analysis may also be required. Whether complicated or uncomplicated, the treatment for acute appendicitis is appendectomy. Appendectomy is the most common emergency surgical operation all over the world [3]. There are two approaches for appendectomy: open appendectomy and laparoscopic appendectomy [5,6,8].

Complicated appendicitis has good prognosis [3] and its mortality rate is less than 1% [2]. Patients who had appendectomy for complicated appendicitis are more likely to develop post-operative complications such as intra-peritoneal abscess or wound infection which are the most common complications [1,2,9]. Patients with these post-operative complications (intra-peritoneal abscess or wound infection) may have fever, lower abdominal pain, constitutional symptoms, diarrhoea or constipation [3]. These post-operative complications may lead or may be associated with longer duration of hospital stay and subsequent higher costs [10].

There are various methods which are applied in order to prevent or to decrease the incidence of these post-operative complications, which are called surgical site infection (intra-peritoneal abscess and wound infection). These methods are: insertion of intra-abdominal drain after the surgery, use of antibiotics, delayed wound closure or the use of laparoscopic technique instead of open technique [1,2,8]. However, insertion of abdominal drain is most familiar than all other methods.

The functions of abdominal drain are: (i) to prevent collection of inflammatory materials, infection debris, bloods, pus and other body fluids at site of surgery [11]. (ii) drainage of already formed collection (iii) by doing first and second function, it may reduce bacterial invasion and colonization at site of surgery and thus decrease the incidence of surgical site infection [12,13]. However, the insertion of abdominal drain may have some drawbacks or disadvantages such as: (i) blockage or obstruction of drain with consequent failure of its function (ii) existence of drain inside the human body may be recognized as foreign body which can initiate inflammatory response and may interfere with surgical site healing (iii) insertion of intra-abdominal drain can increase the duration of patient's stay in the hospital with subsequent extra cost [[12], [13], [14]].

According to the above explanation, the use of intra-abdominal drain after open emergency appendectomy for complicated appendicitis is an issue of great debate. The insertion of drain may reduce or prevent the post -operative surgical site infection (intra-peritoneal abscess and or wound infection) or it may have no effect at all or it may be associated with poor results.

Therefore, the aim of this study was to examine the effectiveness of the use of abdominal drain for preventing or for significantly decreasing the post-operative surgical site infection (intra-peritoneal abscess and or wound infection) of patients with open emergency appendectomy for complicated appendicitis and to find out whether the use of drain is associated with longer hospital stay and higher costs.

## 2 Method

In this retrospective cohort study, files and notes were reviewed for those patients who had open emergency appendectomy for complicated appendicitis and who had already been admitted and discharged from the surgical wards of Kerbala medical university/Imam Hussein medical city hospital/Kerbala/Iraq. This institution is a university hospital and a recognized centre for undergraduate and postgraduate medical studies. The permission and approval for the research was taken from the university. Patients were contacted via phone for their consent for the study according to study protocol. The file and notes of each patient (patient who had open appendectomy for complicated appendicitis) was reviewed retrospectively in terms of three aspects. First, did the patients receive intra-abdominal drain after the surgery or not. Second, whether the patients developed post-operative complications (intra-peritoneal abscess and or wound infection) or not and the third aspect was the length and cost of hospital stay and the mortality outcomes. This work has been reported in line with the STROCSS criteria [15]. The Registration UIN of the study is ACTRN12618000995279.

The patients were included in this research irrespective of their age, sex and race. The study included only the patients who had specific and nearly the same pre, intra and postoperative features in order to avoid any bias. The study included the patients who had perforated appendicitis with localized abscess formation only. All patients received the same course (type, dose, route of administration and duration) of antibiotics pre and post operatively. All patients had the same level of pre, intra and post-operative care. All data had been extracted from patients' files and notes retrospectively. Since all eligible patients, between April/2014 to June/2017, from the hospital records were recruited to this retrospective study (who consented), calculation of sample size was not relevant. A total of 227 eligible patients were identified, which was large enough for testing the hypotheses of this study.

In terms of pre-operative care and features, all participants were seen in accident and emergency (A&E) department for full clinical assessment. The duration of illness before seeking medical advice was nearly the same for all participants. Then they had routine blood tests, urine analysis, imaging and other investigations. After that, they had been admitted to the surgical wards with close monitoring. All of them were nil by mouth and received same type, dose and duration of intravenous fluids, antibiotics, analgesia, anti-pyretic and anti-emetics. They also had the same type, dose, and duration of subcutaneous VTE (Venous thromboembolism) prophylaxis. Participants were selected to have similar levels of physiological derangement pre-operatively (in terms of vital signs parameters) in order to avoid any bias in the study.

Regarding the retrospective review of intra-operative notes; we included only the participants who had open appendectomy for complicated appendicitis. The complicated appendicitis was perforated with localized abscess formation only. Only participants who had similar amounts of intra-peritoneal abscess were included in the study (mild to moderate amount of abscess). All these patients received the same surgical technique, which was open appendectomy with a very good pre-closure surgical wash. All the consultants were at senior level (10 years or more of experience) and the operative time (length of surgery) for all patients was nearly the same. All the included patients were nearly at the same level of physiological derangement intra-operatively. All participants had same type and dose of intra-operative antibiotics and the type of anaesthesia was general for all of them. Moreover, all patients had the same type and size of suture that was used for closure of their wounds.

Post-operatively, the entire participants in the study had the same care. All of them were transmitted to the surgical wards with close monitoring of vital signs; pulse rate, blood pressure, temperature, respiratory rate and O2 saturation. All of the patients had the same type, dose, route of administration and duration of antibiotics. In addition, all of those participants received the same protocol in terms of post-operative instructions, IV fluids, analgesia, blood tests, and when to start oral feeding and mobilization.

The study excluded the following participants: (i) patients with uncomplicated appendicitis (ii) patients with other forms of complicated appendicitis such as gangrenous appendicitis, perforated appendicitis without abscess formation, periappendiceal sub-acute inflammation or others (iii) patients with laparoscopic appendectomy (iv) patients for whom other methods for preventing post-operative surgical site infection, such as different course of antibiotics or delay wound closure etc, applied (v) immune-compromised patients such as DM, HIV or long term steroid therapy or other co-morbidities (vi)patients who received the same course of antibiotics but had antibiotics resistance due to previous exposure. This was obtained from past drug history. (vii) Patients who had a different type or level of pre, intra and post-operative care and characteristics in order to avoid any bias as much as possible. For example: patients who had large amount of abscess or patients who were very unwell or had sever degree of physiological derangement or patients who had long duration of illness or lengthy operation. All those patients were excluded from the study to avoid any bias. (viii) Patients who had their surgeries done by junior consultants (less than 10 years’ experience).

We reviewed the files and notes retrospectively for all patients who were included in the study (according to the inclusion and exclusion criteria above) and divided the participants into two groups: the drainage and non-drainage groups. The drainage group comprised the patients who had open emergency appendectomy for complicated appendicitis (perforated appendicitis with localized abscess) with insertion of abdominal drain after the operation. All patients in this group had the same type and size of abdominal drain. Meanwhile, the non-drainage group consisted of the patients with open emergency appendectomy for complicated appendicitis (perforated appendicitis with localized abscess) but without insertion of abdominal drain after the surgery. The notes and files of both groups had been reviewed retrospectively and a comparison was done between them in terms of the following outcomes:

(i) Development of post-operative intra-peritoneal abscess (abdominal or pelvic). This was assessed and diagnosed depending on clinical features, blood tests and imaging. All patients should require admission and in-patients management if they developed post-operative abscess.
(ii) Development of wound infection. This was either superficial or deep wound infection and was diagnosed by clinical examination, micro-biological tests and sometimes by imaging. This wound infection should be treated on in-patient basis.
(iii) Length of stay in hospital
(iv) Cost of hospital and (v) Mortality rate.

The data was collected on excel spreadsheet. We used Pearson's chi-square test to compare two categorical variables; independent sample *t*-test to compare two normally distributed variables and Mann-Whitney *U* test to compare two variables those were not normally distributed. A 5% level of significance was used for statistical significance.

## 3 Result

The study identified 227 patients with open emergency appendectomy for complicated appendicitis from April/2014–June/2017 fulfilled the inclusion criteria. One hundred and fourteen (114) out of two hundred and twenty seven (227) patients had abdominal drain inserted after the surgery and the others (113 out of 227 patients) did not. The mean age was 31.75 years for the drainage group and 30.77 years for the non-drainage group. For drainage group, 47% were male while for non-drainage group 53% were male. In terms of Body mass Index (BMI), it had been found that 48% of non-drainage group had normal BMI and the others were overweight. Meanwhile, 46% of non-drainage group had normal BMI and the rest (54%) were overweight. Table 1 summarises the patient characteristics.

**Table 1:** Patient characteristics.

Characteristics	Drainage group (114)	Non-drainage group (113)
Gender		
Male	47%	53%
Female	53%	47%
Mean Age	31.75	30.77
BMI		
Normal weight	48%	46%
Over weight	52%	54%

### 3.1 Post-operative complications

It was observed that 50 out of 114 drainage patients (43.9%) experienced post-operative intra-peritoneal abscess while 53 out of 113 non-drainage patients (46.9%) experienced this complication. It was also revealed that 42 out of 114 drainage patients (36.8%) and 38 out of 113 non-drainage patients (33.6%) affected by post-operative wound infection. Pearson's chi-square test was conducted to investigate whether or not there was any association between insertion of abdominal drain and development of post-operative complications (i.e. intra-peritoneal abscess and wound infection). The test result (intra-peritoneal abscess: Pearson's Chi-square = 0.21, *P*-value = 0.65; wound infection: Pearson's Chi-square = 0.26, *P* = 0.61) showed that the insertion of abdominal drain did not have significant effect on development of post-operative complications (intra-peritoneal abscess and wound infection). There was a difference between both groups (drainage and non-drainage groups) in terms of development of post-operative intra-peritoneal abscess and wound infection but this difference was not statistically significant. Table 2 shows the comparisons between drainage and non-drainage group in term of post-operative complications.

**Table 2:** Comparisons between drainage and non-drainage group in term of post-operative complications.

		Drainage	Non-drainage	Total	Pearson's chi-square	*P*-value
Intra-peritoneal abscess	Yes	50	53	103	0.21	0.65
	No	64	60	124		
	Total	114	113	227		
Wound infection	Yes	42	38	80	0.26	0.61
	No	72	75	147		
	Total	114	113	227		

### 3.2 Length of stay in the hospital

It was found that the length of stay in the hospital was longer for the patients in the drainage group than that in the non-drainage group. The mean length of stay for the non-drainage group was 2.12, while it was 4.99 for drainage patients (more than double). Independent sample *t*-test was performed to compare these two means. *P-*value was less than 0.001, which indicates that the patients with drain had statistically significantly longer length of hospital stay compared to patients without drain. Thus, there was a statistically significant difference between drain and non-drainage groups in terms of length of hospital stay. See Table 3 below:

**Table 3:** Comparisons between drainage and non-drainage group in term of length of hospital stay.

		Number of patients	Mean (SD) length of stay, days	*P*-value^a^
Group	Drainage	114	4.99 (1.2)	<0.001
	Non-drainage	113	2.12 (0.9)	

a Independent sample *t*-test.

### 3.3 Hospital cost

To compare the two groups in term of cost, Mann-Whitney *U* test was conducted, see Table 4. The median cost for each drainage patients was $120, while it was $60 for non-drainage patients. The study revealed that the hospital cost for drainage group was statistically significantly higher than that for non-drainage group (*P* < 0.001). This is obviously because the period of stay in the hospital was longer for drainage group than that for non-drainage group.

**Table 4:** Comparisons between drainage and non-drainage group in term of cost of hospital stay.

		Number of patients	Median (I-Q range) cost in dollar	*P*-value^a^
Group	Drainage	114	120 (60)	<0.001
	Non-drainage	113	60 (60)	

a Mann-Whitney *U* Test.

### 3.4 Mortality

There was no loss of life reported in either group.

## 4 Discussion and conclusion

The study found that the insertion of abdominal drainage after open emergency appendectomy for complicated appendicitis had no significant effect on development of post-operative complications, the intra-peritoneal abscess and wound infection. In addition, patients with abdominal drainage had significantly longer period of hospital stay (double) and hence they were costed significantly more. However, there was no mortality found in either group.

Although the insertion of intra-abdominal drain is one of the methods to prevent postoperative complications, the study concluded that the routine insertion of intra-abdominal drain after open appendectomy for complicated appendicitis (perforated appendicitis with localized abscess) does not prevent or significantly decrease the incidence of development of post-operative complications (intra-peritoneal abscess and wound infection).

There are some possible causes which might explain the failure of abdominal drain to prevent or significantly reduce the incidence of post-operative complications. Firstly, abdominal drains may be blocked and occluded by blood, pus, infection debris, fibrin, clots or others. Secondly, the drain may not be enough to drain the whole abdominal cavity [16]. And thirdly, this is a retrospective study and hence may have some limitations to detect any clinically important difference between drainage and non-drainage groups. Although the insertion of drain decreased the intra-peritoneal abscess slightly, it increased the wound infection slightly. However, none of the increase or decrease was statistically significant. Therefore, there is no indication that the routine insertion of intra-abdominal drain after open emergency appendectomy for complicated appendicitis (perforated appendicitis with local abscess formation) can prevent or greatly reduce the possibility of development of post-operative complications (intra-peritoneal abscess and wound infection).

The study found that the period of hospital stay was longer for the drainage group than that for the non-drainage group. The mean length of stay for the non-drainage group was 2.12 days, while it was double (4.99 days) for drainage patients. The reasons behind the longer stay for drainage group may be the time required for drainage process and the possibility of wound infection. The longer hospital stays thus led to higher cost of hospital and it was found statistically significantly higher for drainage group compared to non-drainage group.

By reviewing other studies in the same field, we have found that most of these studies ended up to the same conclusion as of our study and hence supported it. Studies such as de Jesus et al. (2004) and Wang et al. (2011) concluded that routine insertion of abdominal drain after different abdominal surgeries is not necessary [17,18].

Petrowsky et al. found that the use of intra-abdominal drain after open appendectomy for complicated appendicitis does not prevent or reduce the development of post-operative surgical site infection and it might be associated with increased risk of formation of faecal fistula. Therefore, abdominal drain should be avoided after complicated appendicitis [19]. Schlottmann et al. concluded that the placement of intra-abdominal drain may not present benefits and may even lengthen hospital stay [20]. Cheng et al. found that it is not clear whether the routine insertion of intra-abdominal drain after open emergency appendectomy for complicated appendicitis has any effect on the prevention or significant reduction of development of post-operative intra-peritoneal abscess or not. Intra-abdominal drain after open appendectomy for complicated appendicitis may be associated with delayed discharge from the hospital with consequent more cost [3].

Although there are similar studies in this field, our study is different and unique because we included the perforated appendicitis with localized abscess formation only. Meanwhile, most of other similar studies, such as Chang Y et al., included all other types of complicated appendicitis such as gangrenous appendicitis, perforated appendicitis with abscess, perforated appendicitis without abscess, periappendiceal sub-acute inflammation or others [3]. That means our research was more specific and sensitive than other studies in detecting the differences between drainage and non-drainage groups. Therefore, it tested the effectiveness of postoperative abdominal drainage properly. In other words, even if we assume that the futility of abdominal drain in many situations is well established by other randomized studies, this might be true with other types of complicated appendicitis but not with the category in this study. The role of abdominal drainage after open appendectomy for perforated appendicitis with abscess formation is still controversial. And our study found that the use of this approach in this particular situation is not effective to decrease the incidence of postoperative intra-peritoneal abscess and wound infection significantly.

Like any other studies, this study has some limitations which might be avoided in future studies. This was a retrospective study based on single hospital. Although patients with drain had more days of stay in hospital than those without drain, there are many other reasons in addition to drain related problems. Such as past medical history, social factors or others.

Despite few limitations, we can conclude that, the usage of abdominal drain does not help to prevent or significantly reduce the post-operative complications (intra-peritoneal abscess and wound infection) in patients who had emergency open appendectomy for complicated appendicitis (perforated appendicitis with localized abscess formation). Rather, it causes longer hospital stay with subsequent higher cost.

## Provenance and peer review

Not commissioned externally peer reviewed.

## Ethical approval

Permission and approval for research was taken from Kerbala medical university/Imam Hussein medical city hospital/Kerbala/Iraq. Patients were contacted via phone for their consent for the study.

## Sources of funding

None.

## Conflicts of interest

None.

## Research registry number

ACTRN12618000995279.

## Guarantor

Mr Ahmed Kamel Abdulhamid.

## Appendix A Supplementary data

Mutimedia Component Mutimedia Component

Mutimedia Component Mutimedia Component
